# Transforming child and youth mental health care: ACCESS Open Minds New Brunswick in the rural Francophone region of the Acadian Peninsula

**DOI:** 10.1111/eip.12815

**Published:** 2019-06-27

**Authors:** Anik Dubé, Penelopia Iancu, Carole C. Tranchant, Danielle Doucet, Aduel Joachin, Julie Malchow, Sophie Robichaud, Martine Haché, Isabelle Godin, Laure Bourdon, Jimmy Bourque, Srividya N. Iyer, Ashok Malla, Ann M. Beaton

**Affiliations:** ^1^ Faculty of Health Sciences and Community Services, School of Nursing Université de Moncton Moncton New Brunswick Canada; ^2^ ACCESS Open Minds (Pan‐Canadian Youth Mental Health Services Research Network) Douglas Mental Health University Institute Montreal Quebec Canada; ^3^ Faculty of Arts and Social Sciences, School of Social Work Université de Moncton Moncton New Brunswick Canada; ^4^ Faculty of Health Sciences and Community Services, School of Food Science, Nutrition and Family Studies Université de Moncton Moncton New Brunswick Canada; ^5^ Interdisciplinary Research Chair on Children and Youth Mental Health, Faculty of Educational Sciences Université de Moncton Moncton New Brunswick Canada; ^6^ Faculty of Health Sciences and Community Services, School of Psychology Université de Moncton Moncton New Brunswick Canada; ^7^ ACCESS Open Minds‐Esprits ouverts New Brunswick Acadian Peninsula Moncton New Brunswick Canada; ^8^ Department of Psychiatry McGill University Montreal Quebec Canada; ^9^ Prevention and Early Intervention Program for Psychosis (PEPP) Douglas Mental Health University Institute Montreal Quebec Canada

**Keywords:** access, francophone linguistic minority, mental health care, transformation, youth, youth mental health, Canada

## Abstract

**Aim:**

This paper describes how the transformation of youth mental health services in the rural Francophone region of the Acadian Peninsula in New Brunswick, Canada, is meeting the five objectives of ACCESS Open Minds.

**Methods:**

Implementation of the ACCESS Open Minds framework of care in the Acadian Peninsula of New Brunswick began in 2016 at a well‐established volunteer centre and community‐based mental health organization. Through focus groups with youth aged 14 to 22 (n = 13), community mapping was used to describe the youth‐related mental health service transformation, followed by thematic analysis, validation by member checking and triangulation.

**Results:**

Preliminary results show a generally successful implementation of the ACCESS Open Minds model, as evidenced by the transformation of mental health service provision, the enhancement of capacity in human resources and the participation of youth. Transformation was evidenced across the five objectives of mental healthcare of ACCESS Open Minds, albeit to variable extents. Several facilitating factors and challenges are identified based on youths' accounts.

**Conclusions:**

It is possible to successfully implement the ACCESS Open Minds model among francophones living in a minority setting and despite the constraints of a rural area. Most key components of the framework were implemented with high program fidelity. The rural context presents unique challenges that require creative and effective use of resources, while offering opportunities that arise from a culture of resourcefulness and collaboration.

## 1 INTRODUCTION

Access to appropriate services is important for anyone grappling with mental health (MH) challenges. However, for youth who belong to a linguistic and cultural minority group such as the Acadians in New Brunswick, access to appropriate interventions during the early onset of mental illness may be particularly challenging (Dezetter, Beaton, & Bourque, 2016).

Language is a determinant of MH and a low proficiency in the dominant language can create health disparities (Puchala, Leis, Lim, & Tempier, 2013). For many years, MH program coordinators in the Acadian Peninsula have advocated for the importance of community‐based and innovative MH care for youth. Mahmoud, Sers, and Tuite (2016) have noted the scarcity of MH research in this Francophone region. They also called for better quality and greater accessibility of culturally sensitive MH programs.

The pan‐Canadian ACCESS Open Minds (ACCESS OM) project responded to the needs of the youth population. The project aims to transform the way mainstream MH services are delivered to youth aged 11 to 25 years. This transformation model is based on five main objectives of care: (i) early identification of youth in need; (ii) rapid access to mental healthcare; (iii) appropriate care; (iv) continuity of care beyond the age of 18; and (v) youth and family engagement (Malla et al., 2018). This paper aims to describe how the transformation of youth MH services in the Acadian Peninsula is meeting the five objectives of ACCESS OM.

## 2 CONTEXT

**Figure 1:**
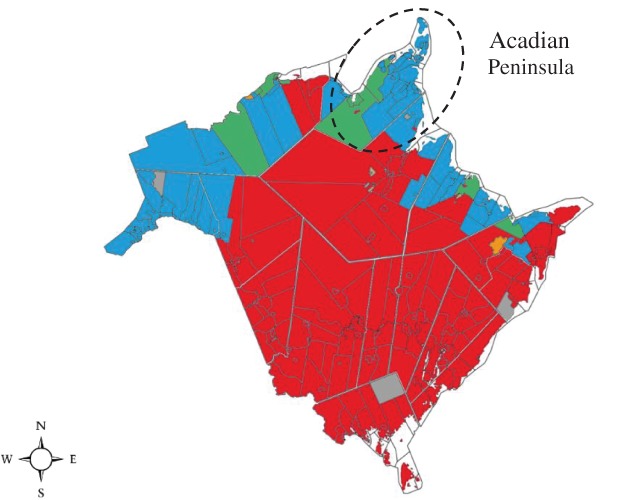
Linguistic map of New Brunswick, Eastern Canada (Statistics Canada, 2006). Red 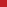: majority English‐speaking, less than 33% French‐speaking. Blue 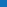: majority French‐speaking, less than 33% English‐speaking. Orange 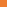: majority English‐speaking, more than 33% French‐speaking. Green 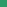: majority French‐speaking, more than 33% English‐speaking. Brown 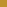: majority allophone (Indigenous languages). Grey 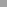: no data available

New Brunswick reflects Canada's diversity: it is rural and urban, officially bilingual and includes different cultures. Many of its population (48%) live in rural areas. In northern New Brunswick, French is predominantly spoken, with fewer than 33% of English speakers (Figure 1).

One of the three sites of ACCESS Open Minds New Brunswick (ACCESS OM NB) is located in the main Francophone Acadian Peninsula (*la Péninsule acadienne*) in northern New Brunswick (Figure 1). It is a rural setting with fishing and agriculture as the dominant industry. The proportion of households with low income (21.4%) is higher than the provincial average (17.2%) (New Brunswick Health Council, 2016a). Its current population is estimated at 50 000 (6.6% of New Brunswick total population), with about 4000 youth aged 10 to 24 years (Statistics Canada, 2016). The average family income in the Peninsula is $31 301CAD after tax for a two persons household (Statistics Canada, 2017).

More children and youth are hospitalized for mental illness over time in the Acadian Peninsula at a rate of 90 to 126 per 10 000 compared to 28 to 51 per 10 000 in other parts of the province. Depressive episodes, stress reactive disorders, behavioural and learning disorders are the leading causes of mental illness admissions in the region (NBHC, 2016b). Among Acadian youth aged 12 to 17 years, 20% report symptoms of depression, while 25% report symptoms of anxiety (NBHC, 2017). Caron and Liu (2010) argue that the proportion of youth in need of MH services is much greater than estimated. In fact, only 8% of total youth received adequate MH treatment in the Acadian Peninsula (Morrison & Peterson, 2017).

## 3 MENTAL HEALTH SERVICES IN NEW BRUNSWICK PRIOR TO ACCESS OM

Comorbidity in youth with mental illness is prevalent in New Brunswick (Caron & Liu, 2010). Further, early onset psychosis is associated with a variety of other MH conditions such as anxiety and addictions, which can disrupt life trajectories and future prospects (Kutcher & McDougall, 2009). Since 2015, New Brunswick has made youth mental healthcare one of its four health priorities (NBHC, 2016a). As a result, new provincial MH policies have focused on integrated service delivery to better coordinate responses to multiple youth‐related problems based on a recovery model of care (Government of New Brunswick, 2018). This provincial momentum provided a leverage for innovative programming such as ACCESS OM (Malla et al., 2018).

Implementation of the ACCESS OM framework in the Acadian Peninsula began in 2017 at the *Centre de bénévolat de la Péninsule acadienne* (CBPA) in Caraquet, a well‐established volunteer centre and community‐based MH organization. Prior to ACCESS OM, youth 13 to 16 years of age had access to a program called Intersection. Although the program touched on some aspects of MH, the purpose of Intersection was the prevention of criminality among youth in the region by engaging them in activities meant to build life‐skills. Staff soon realized that through the Intersection program, youth were often dealing with MH problems. In fact, the majority of youth who participated in the Intersection program lived with undiagnosed and untreated MH issues. At the CBPA, two ACCESS social work Clinicians who hold Bachelor degrees and four youth workers who completed a community integration course provide services designed to meet the five objectives of care. Most clinicians and youth workers that presently work for ACCESS OM NB at the CBPA were also part of the Intersection program, which enabled a natural flow and abundance of admissions at the initial opening.

## 4 COMMUNITY MAPPING

Following the approval of ethics committees, a community mapping of the MH access points in the Acadian Peninsula was conducted. Two separate focus groups were held with youth 14 to 22 years (n = 13), followed by thematic analysis, validation by member checking and triangulation (Braun & Clarke, 2006; Creswell & Poth, 2018). Key informants and youth representatives from different areas of the province provided information to ensure that the research protocol was youth‐appropriate. The purpose of the focus groups was to explore youth perceptions of the different MH access points (people and places). Youth were encouraged to draw or map the different points of access (hospitals, schools, community centres, clinics) and to share their stories. The preliminary findings suggest many of these participants experienced challenges with the standard, heavily regulated and often‐inflexible mental healthcare system in New Brunswick. Some youth also described the formal MH system as disconnected and insufficiently integrated. More importantly, youth explained that they avoided what they described as a dehumanizing formal system that left them feeling traumatized. The following is a description of the ACCESS OM NB transformation in the Acadian Peninsula. Some preliminary findings from the community mapping focus groups has been added to gain a better understanding of the MH transformation.

## 5 MEETING THE OBJECTIVES OF ACCESS OM

### 5.1 Early Identification of youth in need

With respect to early identification of youth in need of MH services, ACCESS OM NB welcomes youth as young as 11 years old and remains flexible with the eligibility criteria for MH assessments and services. Any issue causing distress for youth is a criterion for assessment. Unlike other mental healthcare programs in the region, such as schools and hospitals, youth do not require a predefined MH problem or diagnosis for eligibility. In fact, the ACCESS OM program in the Acadian Peninsula presents fewer barriers for youth to receive faster MH support as compared to other regional services (ie, flexible hours, mobile services). This allows the program to quickly identify and meet with youth experiencing psychological distress before it devolves into a full‐blown mental illness or becomes a crisis.

Collective regional effort, such as inviting other youth‐related programs and MH sectors to the ACCESS OM NB promotional activities (ie, social events, community kitchens or collective cooking classes), has been key to the enhancement of early identification. The program planners make a collective effort not to duplicate or compete with other regional services and programs. They often work in close collaboration with other community sectors (ie, integrated service delivery for children and youth in school, education and early childhood development, social development) to identify the early onset of youth‐related MH problems and identify youth in need.

In our experience, youth who participate in ACCESS OM NB are proud of their program and openly talk about their positive experiences. This creates a sense of ownership, which in turn reduces stigma and labelling associated with the initial willingness to access services. Teachers have shared that youth openly talk about the program at school, which often leads to self‐referrals and early identification.

### 5.2 Rapid access to MH services

Rapid access means that an ACCESS Clinician or a youth worker provides an initial contact and crisis assessment within 72 hours of reaching out for help. The young person will determine the preferred setting for rapid access. In some cases, youth will opt for a meeting in an office space. In other cases outreach is provided and will include virtual contact (eg, text) or a community setting (eg, car). Considering the vastness of the Acadian Peninsula region, outreach represents an important initiative provided by the ACCESS OM team to ensure rapid access to youth‐based care. When the program was launched in the Acadian Peninsula, the flow of youth seeking services increased dramatically. The team quickly realized that the program needed to adapt to meet the target for rapid access. It was decided that when the clinician could not physically meet the youth within 72 hours, the program would ensure that an initial crisis assessment be made by a youth worker by phone or in person. Therefore, the clinician could spend more time with the youth to complete the MH assessments and the admission process (eg, bio‐psycho‐social and research assessments).

Due to a shared youth‐centred approach, the ACCESS OM NB site and other community and government services (ie, Child and Youth MH services) in the region developed an informal partnership to meet the 72 hour requirement. The ACCESS OM approach is flexible, allowing the on‐call clinician or youth worker who receives a call from a youth in crisis, to assess the situation and respond accordingly. For instance, this may require the clinician or youth worker to advocate on behalf of the youth to receive an initial assessment from a government‐based MH service and accompany the youth at every stage of this process. Furthermore, if other community organizations or government services recognize the need for a 72 hour assessment, and feel that the ACCESS OM team is better suited to rapidly meet the needs of the youth, the youth will be referred to the ACCESS Clinician. Such close collaboration between the ACCESS OM team with government and community sectors represents a major change to MH care for youth.

The importance of rapid access was mentioned by many youth who participated in the community mapping focus groups. They explained that flexibility was one of the most influential features of rapid access. Youth added that when a crisis occurs, the clinician or youth worker will come to them, which is very beneficial for a youth who lives in a more remote area.

### 5.3 Appropriate care

Access to evidence‐informed intervention through an integrated mental healthcare approach is a priority. The ACCESS OM staff offer youth‐related MH counselling services in an informal and comfortable setting, oftentimes outside the conventional “office space.” Youth are offered different options for access to mental healthcare: (i) counselling with a clinician; (ii) counselling with a clinician and follow‐up care with a youth worker; (iii) counselling with a clinician, follow‐up care with a youth worker and program of activities; (iv) program of activities if youth already has adequate mental healthcare (eg, school, clinic) in place but still wants to participate in activities to enhance life skills.

To adequately meet the needs of youth, the team developed an evidence‐informed individual counselling therapy and skill building program in a mobile “safe space” setting (ie, text, phone, coffee shop), where clinicians and youth workers will go to the youth in need for assessments, counselling and follow‐up care. At first glance, the program of activities might appear to focus on leisure. However, they are all framed to build upon specific skills, such as anxiety/depression awareness, counselling, relaxation strategies, healthy relationships, self‐esteem building, social skills, job readiness, youth mentorship skills, assertiveness skill building, crisis and suicide prevention strategies.

The ACCESS OM NB site does not employ a clinical psychologist, sexologist or psychiatrist to offer more complex psychotherapy and behavioural interventions. Therefore, a member of the site team (clinician or youth worker) will initially meet the youth to assess the crisis level and refer to a more specialized program (eg, emergency department, psychiatrist) for further complex mental healthcare, such as an untreated episode of psychosis. Long wait time for MH specialized care is a major challenge for many youth and unfortunately creates further MH complications that often can be prevented with rapid access to care. What has been key to the success of ACCESS OM is its ability to remain with the youth during their MH crisis continuum, from the initial contact to the assessment of MH, the management of immediate care if needed (emergency department) and the coordination of long‐term services with appropriate specialist.

### 5.4 Continuity of care

Youth transitioning to the adult MH program was problematic in the region, as the waiting list was long and young adults would go several months to a year without adequate care. The continuity of care objective is particularly important at the CBPA, as it responds to a critical need for adequate care in the region and allows for a better transition of MH services for young adults up to 25 years old. At the inception of ACCESS OM NB, the program coordination and service providers informed youth‐related sectors (youth protection services, clinical psychologists and psychiatrists) about the availability of MH services for young adults transitioning from the youth program to the adult program, which has a significant waitlist and long wait time for admission. There was a natural transition with ACCESS, as the older youth had previously built a trusting relationship with the intersection program clinicians and youth workers.

For youth living with a MH issue, the transition to adulthood can be a challenge. The ACCESS Clinician and youth workers at the CBPA recognize the importance of continuity of care beyond 18 years of age. They offer counselling and care that is required by the young person and designed to help them gain different life skills (eg, planning budget, searching for employment). Along with appropriate MH services, ACCESS Clinician and youth workers collaborate with other community services to ensure that all youths who reach 18 continue to have access to the care they need.

### 5.5 Youth and family engagement

The Youth Advisory Committee offers youth a voice in the governance structure of ACCESS OM NB at the CBPA. They are directly part of the decision‐making process related to programming and intervention. Peer support is also an important component of ACCESS OM. Yet, at the onset, the community clinicians and youth workers were reluctant to assign youth mentors to incoming youth without providing them with any support or training. Therefore, the ACCESS Clinicians and the youth workers specifically choose the youth mentors according to their life trajectories, their MH outcome, and their capacity to mentor new youth in search of mental healthcare. Furthermore, clinicians and youth workers aid youth in building their mentorship skills to help welcome new and younger youth to the program and facilitate engagement activities. The ACCESS OM NB Family‐Caregivers Advisory Committee has been more challenging to establish. The scarcity of parent participation is noteworthy but not entirely surprising. Most families and youth have complex issues, histories and present challenges affecting their ability to connect. Indeed, the ACCESS OM project is relatively new to the community and mainstream mental healthcare services generally do not emphasize family engagement. According to the ACCESS OM team, young people search for a meaningful and secure relationship with youth workers. This is a priority for the ACCESS OM team at the CBPA and the key to keeping youth engaged.

During the community mapping focus groups, preliminary findings suggest that some youth do not want their family members involved in their mental healthcare, as some parents may also be affected by mental illnesses or other issues or might contribute to some of the difficulties experienced by youth. Although many youth shared that they did not have close relationships with their family, they still expressed a hope to become closer one day. Youth also identified people or groups that represented a “family substitute” in their lives because they played an influential and beneficial role regarding their mental healthcare. The fact that families were not mentioned by participating youth in the focus groups as an important support for mental healthcare does not mean that families are disengaged. Further research is underway to better understand the parent's perception and needs regarding youth MH in the region.

## 6 WHAT IS SPECIAL ABOUT THIS SITE?

A unique feature of the ACCESS OM NB site at the CBPA is its ability to combine the Intersection and the ACCESS OM programming to meet the MH needs of youth. The ACCESS OM objectives also build on an existing MH platform of integrated service delivery recently established by the province to improve access to MH related services.

Despite the challenges of remoteness and limited resources, this site heavily draws on a culture of resourcefulness, capacity building and collaboration, both within the community and with outside service providers. Important bottlenecks such as limited number of clinicians, lack of integration in service delivery, and restrictive eligibility criteria have been identified and rapidly addressed in order to reach out to more youth in need. Likewise, the site developed a mobile “safe space” venue for assessment and counselling, enabling rapid access and early identification, thus minimizing some important gaps of MH services such as difficult access, stigma and long wait times for young adults. The program also actively invites youth to participate and be part of the decision‐making mental healthcare processes as well as promotional activities that do not focus solely on MH. Finally, yet importantly, the service providers at this site consider establishing trust in ways that are meaningful to youth. The ACCESS Clinician and youth worker will adapt and meet youth at a local coffee shop if needed to complete assessment or offer counselling.

A number of facilitators (eg, key champions, building of trust) identified by youth may contribute to the development of protective factors that help build resilience in youth, which in turn contributes to improved MH (NBHC, 2016c). These protective factors include an ability to solve problems without harming oneself or others, knowledge of where to go in the community to get help, people to look up to, caregivers who are well‐informed about the youth, the support of friends during difficult times, as well as opportunities to develop useful skills. The development of these protective factors is essential to sustain the beneficial impacts of programs such as ACCESS OM on youth MH and well‐being.

## 7 SUSTAINABILITY

A dialogue is underway between the government of New Brunswick decision‐makers, ACCESS OM NB site leads, and stakeholders, such as family members and First Nations community representatives, to negotiate the terms of integration of ACCESS OM NB within the provincial Network of Excellence. The Network of Excellence is a government strategy whereby a collaboration between Social Development, Education and Early Childhood Development, Health, Justice and Public Safety, and Regional Health Authorities allows for the planning and implementation of a coherent approach to service delivery designed to support children and youth who experience addictions and MH challenges. Some of the key issues under discussion include governance (eg, collaborative leadership), programming and intervention (eg, definition of respective roles in continuum of service), communication and training (eg, sharing of information).

## 8 COMMUNITY IMPACT

A fundamental transformation of youth MH services in the Acadian Peninsula has been underway since 2016, due to the launch of ACCESS OM. As a result, the five objectives of the ACCESS OM framework of care have been reached, albeit to variable extents. Several facilitating factors and challenges were identified based on youth accounts. Challenges such as stigma related to MH and limited family engagement will require further consideration by the ACCESS OM NB team.

It was further noted that many facilitators in the ACCESS OM transformation enhance the deployment of key principles of mental healthcare, and contribute to the development of protective factors. In turn, these protective factors promote resilience in youth and improve MH outcomes and health trajectory. Overall, the preliminary results obtained at this site show that it is possible to implement the ACCESS OM framework in a rural area among francophones living in a minority context. The rural context presents unique challenges that require a strategic use of community assets and socio‐cultural strengths.

## 9 CHALLENGES

Youths' accounts in the Acadian Peninsula suggest that stigma, confidentiality and the delay of some referrals are important factors hindering the early identification of MH issues. This supports previous perspectives on youth MH (Kutcher & McDougall, 2009). It is also noteworthy that youth in this region did not identify family members as sources of support in times of crisis or for help with other matters regarding MH. Youth also identified gaps in the current traditional MH system, such as administrative constraints, delays, lack of integration and inflexible hours, which seem to be major hurdles in meeting their needs. These findings helped the ACCESS OM NB team to reflect critically on innovative approaches to reach all youth in need and find ways to better connect with the traditional MH services. The perspectives of other stakeholders, including family members, carers and other community members will be considered in the near future to gain a comprehensive understanding of the ongoing transformation of youth mental healthcare in the Acadian Peninsula and its impact in the community.
